# Antimicrobial Metabolites against Methicillin-Resistant *Staphylococcus aureus* from the Endophytic Fungus *Neofusicoccum australe*

**DOI:** 10.3390/molecules26041094

**Published:** 2021-02-19

**Authors:** Melissa M. Cadelis, Soeren Geese, Benedict B. Uy, Daniel R. Mulholland, Shara J. van de Pas, Alex Grey, Bevan S. Weir, Brent R. Copp, Siouxsie Wiles

**Affiliations:** 1 School of Chemical Sciences, University of Auckland, Private Bag 92019, Auckland 1142, New Zealand; b.copp@auckland.ac.nz; 2 Bioluminescent Superbugs Lab, School of Medical Sciences, University of Auckland, Private Bag 92019, Auckland 1142, New Zealand; s.geese@auckland.ac.nz (S.G.); b.uy@auckland.ac.nz (B.B.U.); d.mulholland@auckland.ac.nz (D.R.M.); s.vandepas@auckland.ac.nz (S.J.v.d.P.); alex.grey@auckland.ac.nz (A.G.); 3 Manaaki Whenua-Landcare Research, Private Bag 92170, Auckland 1142, New Zealand; WeirB@landcareresearch.co.nz

**Keywords:** antimicrobial, natural product, fungi, MRSA, naphthoquinone dimer

## Abstract

Antimicrobial bioassay-guided fractionation of the endophytic fungi *Neofusicoccum australe* led to the isolation of a new unsymmetrical naphthoquinone dimer, neofusnaphthoquinone B (**1**), along with four known natural products (**2**–**5**). Structure elucidation was conducted by nuclear magnetic resonance (NMR) spectroscopic methods, and the antimicrobial activity of all the natural products was investigated, revealing **1** to be moderately active towards methicillin-resistant *Staphylococcus aureus* (MRSA) with a minimum inhibitory concentration (MIC) of 16 µg/mL.

## 1. Introduction

In 2014, the World Health Organization (WHO) described how drug-resistant microbes are present in every region of the world [1]. The report concluded that within a decade, antimicrobial resistance will make routine surgery, organ transplantation, and cancer treatment life-threateningly risky [1]. Key to managing this crisis is to boost the number of new antibiotic classes reaching the clinic [2,3]. The International Collection of Microorganisms from Plants (ICMP), curated by the Crown Research Institute Manaaki Whenua, has over 10,000 fungal cultures derived from plants and soil from Aotearoa New Zealand and the South Pacific. The collection has a great diversity of fungal species, host substrates, and collection localities, with the earliest cultures dating from the early 1960s [4]. 

**Figure 1:**
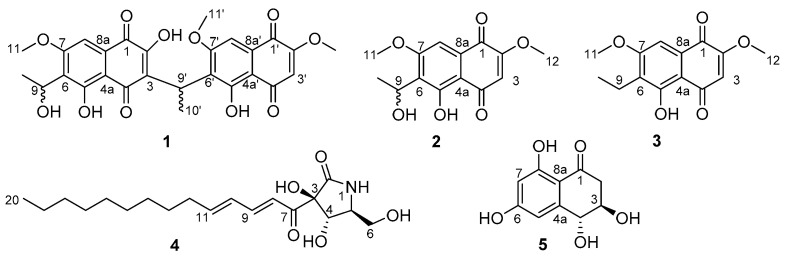
Structures of isolated compounds **1**–**5**.

**Figure 2:**
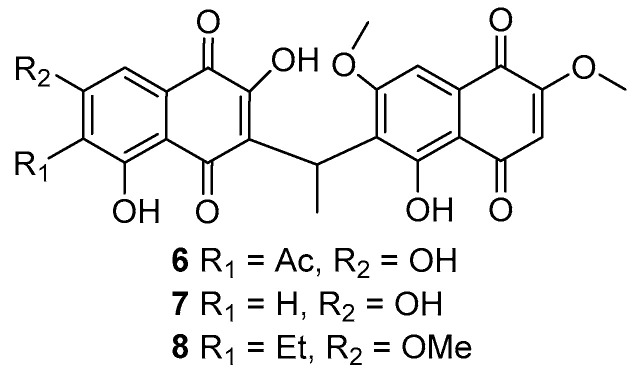
Structures of known naphthoquinone dimers **6**–**8**.

In our search for new bioactive compounds, we began screening ICMP isolates for antibacterial activity [5] against members of the WHO’s “priority pathogens” list [6], including *Escherichia coli*, *Klebsiella pneumoniae*, and *Staphylococcus aureus*. Bioassay directed investigation of an isolate of the endophytic fungi, *Neofusicoccum australe*, led to the isolation of a novel unsymmetrical naphthoquinone dimer **1** and four other known natural products (**2**–**5**) (Figure 1). Other reported unsymmetrical naphthoquinone dimers include kirschsteinin (**6**) isolated from *Kirschsteiniothelia* sp. [7], deacetylkirschsteinin (**7**) isolated from *Phaeosphaeria* sp. [8] and neofusnaphthoquinone A (**8**) isolated from *Neofusicoccum australe* [9] (Figure 2). Structure verification of the four known natural products, which included two naphthalene monomers (**2** and **3**) [10], pramanicin A (**4**) [11] and 4-hydroxyscytalone (**5**) [8], was conducted by comparison of ^1^H NMR data with those reported in the literature. Herein, the isolation, structure elucidation and bioactivity of neofusnaphthoquinone B (**1**) are described.

## 2. Results and Discussion

**Figure 3:**
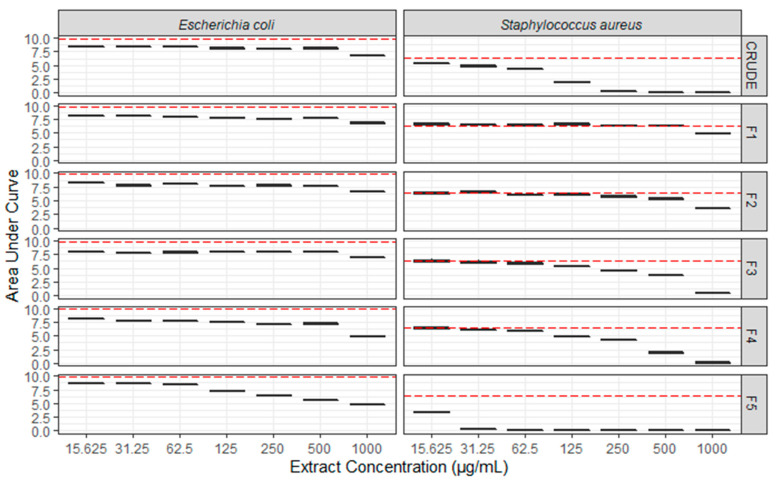
Antibacterial activity of fungal crude extracts and fractions against *E. coli* ATCC 25922 and *S. aureus* ATCC 29213. Activity is shown as box-whisker plots of Area Under Curve (AUC) values of changes in optical density at 600 nm. Prior to calculation of AUC, all optical density values were adjusted to account for the absorbance of the fraction alone by subtracting the values from time zero. Dotted line shows AUC values for bacteria without fungal extracts. Raw data are available at https://doi.org/10.17608/k6.auckland.11868675 (accessed on 19 February 2021).

Antimicrobial screening of ICMP isolates against antibiotic-sensitive and antibiotic-resistant strains of *Escherichia coli*, *Klebsiella pneumoniae* and *Staphylococcus aureus* identified *Neofusicoccum australe* as a hit (see supporting information). During the testing of fungal crude extracts against *E. coli* ATCC 25922 and *S. aureus* ATCC 29213, activity was observed primarily against *S. aureus* (Figure 3). Initial fractionation of the crude extract was conducted by C_8_ reversed-phase column chromatography, eluting with a gradient of H_2_O/MeOH, to afford five fractions (F1–F5). Antimicrobial testing of F1–F5 against the same two microbes identified *S. aureus* activity in F3, F4 and F5 (Figure 3). The most potent inhibitory activity was observed in F5, which displayed inhibition of *S. aureus* ATCC 29213 in a dose-dependent manner with a minimum inhibitory concentration (MIC) of 32 µg/mL. Further purification of combined F4 and F5 led to the isolation of compounds **1**–**4**, while purification of F3 afforded compound **5**.

**Figure 4:**
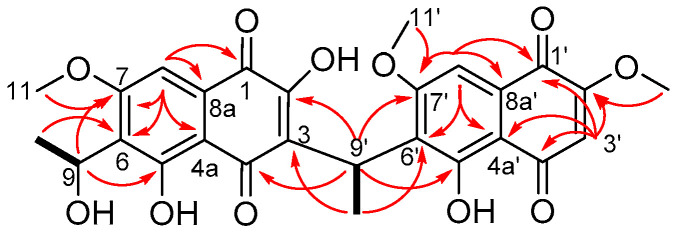
Selected COSY (solid line) and HMBC (arrows) correlations for **1**.

Compound **1** was isolated as the sodiated adduct with a molecular formula C_27_H_24_O_11_ by high resolution ESI mass spectrometry (HRESIMS) *m/z* 547.1203 [M + Na]^+^ (calcd. 547.1211). The ^1^H-NMR spectrum (Figure S1) showed the presence of 24 protons, which included three aromatic signals at δ_H_ 6.24 (s, H-3′), 7.11 (s, H-8) and 7.12 (s, H-8′); three methoxy signals at δ_H_ 3.86 (s, H_3_-11′, H_3_-12′) and 3.92 (s, H_3_-11); two methyl signals at δ_H_ 1.41 (d, *J* = 6.7 Hz, H_3_-10) and 1.60 (d, *J* = 7.0 Hz, H_3_-10′); two methine signals at δ_H_ 4.80 (q, *J* = 7.0 Hz, H-9′) and 5.20 (dq, *J* = 7.0, 6.7 Hz, H-9); and four hydroxyl signals at δ_H_ 4.61 (d, *J* = 7.0 Hz, 9-OH), 10.89 (br s, 2-OH), 12.95 (s, 5′-OH) and 13.38 (br s, 5-OH) (Table 1). The ^13^C-NMR spectrum (Figure S2) identified the presence of 27 carbons, which included four quinone carbons (δ_C_ 178.7, 180.7, 189.9, 190.3), six oxygenated sp^2^ carbons (δ_C_ 159.6, 159.7, 160.2, 160.7, 161.3, 162.5), three protonated sp^2^ carbons (δ_C_ 102.1, 102.9, 109.3), seven other sp^2^ quaternary carbons (δ_C_ 108.3, 108.6_,_ 123.5, 126.6, 126.8, 129.6, 130.0), four sp^3^ oxygenated carbons (δ_C_ 56.2, 56.3, 56.8, 60.6) and three other sp^3^ carbons (δ_C_ 17.6, 21.7, 27.5). Comparison of the NMR data of **1** with kirschsteinin (**6**) [8], another unsymmetrical naphthoquinone, which has an acetyl moiety instead of a hydroxyethyl moiety as well as the presence of an additional methoxy group, showed similarities. Only two COSY cross correlations were observed, one between H-9′ (δ_H_ 4.80, q, *J* = 7.0 Hz) and H_3_-10′ (δ_H_ 1.60, d, *J* = 7.0 Hz) on the ethylidene linker and the other between H-9 (δ_H_ 5.20, dq, *J* = 7.0, 6.7 Hz) and H_3_-10 (δ_H_ 1.41, d, *J* = 6.7 Hz) on the hydroxyethyl fragment (Figure 4). Key HMBC correlations were observed (Figure 4) between H_3_-10′ (δ_H_ 1.60, d, *J* = 7.0 Hz) and C-3 (δ_C_ 123.5) and C-6′ (δ_C_ 126.8), which identified connectivity between the two naphthoquinone fragments with additional correlations between H_3_-10 (δ_H_ 1.41, d, *J* = 6.7 Hz) and C-6 (δ_C_ 126.6) and between H-9 (δ_H_ 5.20, dq, *J* = 7.0, 6.7 Hz) and C-5 (δ_C_ 159.7), showing connectivity of the hydroxyethyl fragment to the naphthoquinone ring. This confirmed the chemical structure of neofusnaphthoquinone B (**1**) as shown. 

The stereochemistry at C-9 and C-9′ was not assigned, as neofusnaphthoquinone B (**1**) was optically inactive and exhibited no electronic circular dichroism (ECD) absorption curves. Attempts were made to determine the configuration at C-9 using the chiral derivatising agent α-methoxyphenylacetic acid (MPA) to prepare diastereomeric esters at C-9 [12]. Treatment of **1** with (*S*)-MPA in the presence of EDC.HCl and DMAP overnight resulted in degradation products, suspected to be due to reaction of MPA with the phenols present in **1** (data not shown). Thus, attempts were made to first protect the phenols using TMS-diazomethane before reaction with (*S*)-MPA [13,14]. Reaction of **1** with TMS-diazomethane with DIPEA for 15 h resulted in degradation products as did the reaction for 6 h (data not shown). 

The isolation of **1** as a racemic mixture is not uncommon for this class of natural products [8,9]. To the best of our knowledge, this is the fourth example of an unusual class of natural products which contain two naphthoquinone subunits connected in a head-to-tail fashion by an ethylidene linker [7,8,9]. The known compounds were identified as 6-(1-hydroxyethyl)-2,7-dimethoxyjugalone (**2**) [10], 6-(1-ethyl)-2,7-dimethoxyjugalone (**3**) [10], pramanicin A (**4**) [11] and (3*S*,4*S*)-4-hydroxyscytalone (**5**) [8]. 

The antimicrobial activity of **1**–**3** and **5** was evaluated against a panel of Gram-positive (methicillin-resistant *S. aureus*) and Gram-negative (*Pseudomonas aeruginosa*, *E. coli*, *K. pneumoniae* and *Acinetobacter baumanii*) bacteria and two fungal strains (*Candida albicans* and *Cryptococcus neoformans*) (Table 2). The antimicrobial activity of pramanicin A (**4**) was not investigated in the present study due to lack of sample; however, the antifungal activity of the natural product has been previously reported [15]. Both neofusnaphthoquinone B (**1**) and monomer **2** exhibited activity against MRSA with **1** exhibiting more potent activity than **2**. Interestingly, during initial ZOI screening, ICMP 21498 (see supporting information) appears equally potent against both *S. aureus* and *E. coli* isolates; however, this indiscriminate killing does not appear to have persisted past extraction. It is, therefore, a possibility that several compounds may be responsible for the antibacterial activity of ICMP 21498 and the other compound(s) were not extracted. 

Both natural products **1** and **2** were also screened for cytotoxicity against human embryonic kidney cells (HEK293) and red blood cell haemolytic properties (Table 3). No cytotoxicity or haemolytic activity was observed for both **1** and **2**, which, combined with the moderate antimicrobial activity against MRSA, makes these compounds of interest. 

## 3. Materials and Methods

### 3.1. General Experimental Procedures

Melting points were measured on a Reichert melting point apparatus (Reichert, Vienna, Austria). Infrared spectra were recorded on a Perkin Elmer Spectrum 100 Fourier Transform infrared spectrometer (PerkinElmer, Boston, MA, USA) equipped with a universal ATR accessory. V_max_ are expressed with units of cm^−1^. Ultraviolet-visible spectra were acquired using a UV-2101 PC UV-Vis scanning Shimadzu spectrophotometer (Shimadzu, Kyoto, Japan) with a pair of 1 cm path length rectangular quartz cuvettes (3 mL, type 3) to measure λmax and log ε expressed in units of nm. NMR spectra were recorded using a Bruker Avance DRX-400 spectrometer or an Avance III-HD 500 spectrometer (Bruker, Karlsruhe, Germany) operating at 400 or 500 MHz for ^1^H nuclei and 100 or 125 MHz for ^13^C nuclei utilizing standard pulse sequences at 298 K. Chemical shifts are expressed in parts per million (ppm) relative to the residual non-deuterated solvent in ^1^H-NMR and to deuterated solvent in ^13^C-NMR (CD_3_OD: δ_H_ 3.31, δ_C_ 49.0; DMSO-*d*_6_: δ_H_ 2.50, δ_C_ 39.52). For ^1^H-NMR, the data are quoted as position (δ), relative integral, multiplicity (s = singlet, d = doublet, t = triplet, q = quartet, dd = doublet of doublets, dq = doublet of quartets, m = multiplet, br = broad), coupling constant (*J*, Hz) and assignment of the atom. The ^13^C-NMR data are quoted as position (δ) and assignment of the atom. High resolution mass spectra were recorded on a Bruker micrOTOF QII (Bruker Daltonics, Bremen, Germany). Analytical thin layer chromatography (TLC) was carried out on 0.2 mm thick plates of DC-plastikfolien Kieselgel 60 F254 (Merck, Munich, Germany). Reversed-phase column chromatography was carried out on C_8_ support with a pore size of 40–63 µm (Merck, Munich, Germany). Gel filtration chromatography was carried out on Sephadex LH-20 (Pharmacia). Flash chromatography was carried out on Diol-bonded silica with a pore size of 40–63 microns (Merck, Munich, Germany). Analytical reversed-phase HPLC was run on a Dionex UltiMate 3000RS system (Waltham, MA, USA) using a C_8_ column (3 µm Econosphere Rocket, 7 × 33 mm) (Grace, Columbia, MA, USA) and eluting with a linear gradient of H_2_O (0.05% TFA) to MeCN over 20 min at 2 mL/min. All solvents used were of analytical grade or better and/or purified according to standard procedures. Chemical reagents used were purchased from standard chemical suppliers and used as purchased. 

### 3.2. Fungal Material

The ascomycete fungus *Neofusicoccum australe* (ICMP 21498) was isolated from diseased grapevines in New Zealand [16]. The isolate was identified based on a match of the sequence of the fungal barcode locus ITS (GenBank: MT107904) to reference specimens.

### 3.3. Fermentation, Extraction and Isolation

Cultures of *Neofusicoccum australe* were grown on forty-two PDA plates at room temperature for 3 weeks and freeze-dried. The dry cultures (23.74 g, dry weight) were extracted with MeOH (2 × 800 mL) for 4 h followed CH_2_Cl_2_ (800 mL) overnight. Concentration of the combined organic extracts under reduced pressure afforded an orange oil (1.69 g). The crude extract was subjected to C_8_ reversed-phase column chromatography eluting with gradient H_2_O/MeOH to afford five fractions (F1–F5). Purification of F3 (3:1, H_2_O/MeOH) by Sephadex LH20 (MeOH) afforded 4-hydroxyscytalone (**5**) (4.60 mg). F4 and F5 were combined and purified by Sephadex LH20 (MeOH) to afford four fractions (A1–A4). Further purification of A4 by diol-bonded silica gel column chromatography (gradient *n*-hexane/EtOAc) afforded 8 fractions (B1–B8). Fraction B2 was triturated with CH_2_Cl_2_ to give 6-(1-ethyl)-2,7-dimethoxyjugalone (**3**) (2.18 mg) as an orange solid. Fractions B3 and B4 were subjected to diol-bonded silica gel column chromatography (gradient *n*-hexane/EtOAc) to afford 6-(1-hydroxyethyl)-2,7-dimethoxyjugalone (**2**) (1.05 mg) as an orange solid. Fraction B7 was triturated with CH_2_Cl_2_ to afford neofusnaphthoquinone B (**1**) (5.30 mg) as an orange solid. Fraction B8 afforded pramanicin A as a pale orange solid (**4**) (0.90 mg).

*Neofusnaphthoquinone B* (**1**): orange solid; [α]_D_^21^ = 0.0 (*c* = 0.09, CH_2_Cl_2_); UV (MeOH) λ_max_ [log ε] 415.5 (4.30), 312.5 (4.65), 265.0 (4.96), 219.5 (5.11), 204.0 (5.05); m.p. 213–215 °C; IR (ATR) ν_max_ 3374, 2918, 2851, 1625, 1610, 1586, 1346, 1306, 1243, 1210, 1111, 857, 789 cm^−1^; ^1^H-NMR (DMSO-*d*_6_, 500 MHz) δ 12.95 (1H, s, 5′-OH), 10.89 (1H, br s, 2-OH), 7.12 (1H, s, H-8′), 7.11 (1H, s, H-8), 6.24 (1H, s, H-3′), 5.20 (1H, dq, *J* = 7.0, 6.7 Hz, H-9), 4.80 (1H, q, *J* = 7.0 Hz, H-9′), 4.61 (1H, d, *J* = 7.0 Hz, 9-OH), 3.92 (3H, s, H_3_-11), 3.86 (6H, s, H_3_-11′, H_3_-12′), 1.60 (3H, d, *J* = 7.0 Hz, H_3_-10′), 1.41 (3H, d, *J* = 6.7 Hz, H_3_-10); ^13^C-NMR (DMSO-*d*_6_, 125 MHz) δ 190.3 (C-4′), 189.9 (C-4), 180.7 (C-1), 178.7 (C-1′), 162.5 (C-7′), 161.3 (C-7), 160.7 (C-2′), 160.2 (C-5′), 159.7 (C-5), 159.6 (C-2), 130.0 (C-8a’), 129.6 (C-8a), 126.8 (C-6′), 126.6 (C-6), 123.5 (C-3), 109.3 (C-3′), 108.6 (C-4a), 108.3 (C-4a’), 102.9 (C-8′), 102.1 (C-8), 60.6 (C-9), 56.8 (C-12′), 56.3 (C-11′), 56.2 (C-11), 27.5 (C-9′), 21.7 (C-10), 17.6 (C-10′); (+)-HRESIMS *m/z* 547.1203 [M + Na]^+^ (calcd. for C_27_H_24_NaO_11_, 547.1211).

*6-(1-Hydroxyethyl)-2,7-dimethoxyjugalone* (**2**): orange solid; [α]_D_^23^ = 0.0 (*c* = 0.07, CH_2_Cl_2_) (lit optically inactive [10]); m.p. 202–204 °C (lit 201–204 °C [10]); ^1^H-NMR (CD_3_OD, 400 MHz) δ 7.26 (1H, s, H-8), 6.15 (1H, s, H-3), 5.38 (1H, q, *J* = 7.2 Hz, H-9), 3.93 (3H, s, H_3_-11), 3.91 (3H, s, H_3_-12), 1.72 (3H, t, *J* = 7.2 Hz, H_3_-10); (+)-HRESIMS *m/z* 301.0687 [M + Na]^+^ (calcd. for C_14_H_14_NaO_6_, 301.0680).

*6-(1-Ethyl)-2,7-dimethoxyjugalone* (**3**): orange solid; m.p. 187–189 °C (lit 186–188 °C [10]); ^1^H NMR (CD_3_OD, 400 MHz) δ 7.30 (1H, s, H-8), 6.18 (1H, s, H-3), 4.00 (3H, s, H_3_-11), 3.93 (3H, s, H_3_-12), 2.76 (2H, q, J = 7.3 Hz, H_2_-9), 1.13 (3H, t, J = 7.3 Hz, H_3_-10); ^13^C-NMR (CD_3_OD, 100 MHz) δ 191.7 (C-4), 180.8 (C-1), 163.7 (C-7), 162.3 (C-2), 161.3 (C-5), 131.5 (C-8a), 128.0 (C-6), 110.3 (C-4a), 110.2 (C-3), 103.4 (C-8), 57.2 (C-12), 56.6 (C-11), 17.0 (C-1′), 13.2 (C-2′); (+)-HRESIMS *m/z* 285.0729 [M + Na]^+^ (calcd. for C_14_H_14_NaO_5_, 285.0733).

*Pramanicin A* (**4**): pale orange solid; [α]_D_^20^ = −35 (*c* = 0.09, MeOH) (lit [α]_D_^25^ = −35 (*c* = 0.21, MeOH) [15]); m.p. 112–114 °C (lit 110–113 °C [15]); ^1^H-NMR (CD_3_OD, 400 MHz) δ 7.30–7.24 (1H, m, H-9), 6.73 (1H, d, *J* = 15.2 Hz, H-8), 6.31–6.28 (2H, m, H-10, H-11), 4.14 (1H, d, *J* = 6.6 Hz, H-4), 3.80 (1H, dd, *J* = 11.0, 2.4 Hz, H_2_-6_A_), 3.54 (1H, dd, *J* = 11.0, 5.5 Hz, H_2_-6_B_), 3.50 (1H, dd, *J* = 5.5, 2.4 Hz, H-3), 2.23–2.18 (2H, m, H_2_-12), 1.47–1.42 (2H, m, H_2_-13), 1.32–1.26 (12H, m, H_2_-14, H_2_-15, H_2_-16, H_2_-17, H_2_-18, H_2_-19), 0.89 (3H, t, *J* = 6.6 Hz, H_3_-20); ^13^C-NMR (CD_3_OD, 100 MHz) δ 198.8 (C-7), 175.3 (C-2), 148.7 (C-11), 145.6 (C-9), 130.5 (C-10), 124.2 (C-8), 88.1 (C-3), 79.0 (C-4), 62.1 (C-6), 60.4 (C-5), 34.2 (C-12), 33.1 (C-18), 30.7 (C-14/C-15/C-16/C-17), 30.6 (C-14/C-15/C-16/C-17), 30.5 (C-14/C-15/C-16/C-17), 30.3 (C-14/C-15/C-16/C-17), 29.9 (C-13), 23.7 (C-19), 14.5 (C-20) [^1^H and ^13^C-NMR data agreed with the literature [11]]; (+)-HRESIMS *m/z* 376.2077 [M + Na]^+^ (calcd. for C_19_H_31_NNaO_5_, 376.2090).

*(3S,4S)-4-Hydroxyscytalone* (**5**): colourless gum; [α]_D_^20^ = +43.4 (*c* = 0.03, MeOH) (lit [α]_D_^26^ = +57.92 (*c* 0.07, MeOH) [8]); ^1^H-NMR (CD_3_OD, 400 MHz) δ 6.62 (1H, d, *J* = 2.4 Hz, H-5), 6.18 (1H, d, *J* = 2.4 Hz, H-7), 4.50 (1H, d, *J* = 7.4 Hz, H-4), 4.00–3.96 (1H, m, H-3), 2.96 (1H, dd, *J* = 17.0, 4.3 Hz, H_2_-2_A_), 2.63 (1H, dd, *J* = 17.0, 8.8 Hz, H_2_-2_B_); ^13^C-NMR (CD_3_OD, 100 MHz) δ 201.2 (C-1), 167.8 (C-8), 166.4 (C-6), 148.4 (C-4a), 110.2 (C-8a), 108.7 (C-5), 102.6 (C-7), 73.7 (C-4), 71.8 (C-3), 44.3 (C-2); (+)-HRESIMS *m/z* 233.0424 [M + Na]^+^ (calcd. for C_10_H_10_NaO_5_, 233.0420).

### 3.4. Antimicrobial Activity of Fungal Cultures

Pre-screening of the ICMP fungal cultures for antimicrobial activity involves briefly growing the cultures on potato dextrose agar (PDA) before small wells are cut into the agar and each well inoculated with 5 × 10^6^ colony forming units of luciferase-tagged derivatives of *Escherichia coli*, *Klebsiella pneumoniae*, and *Staphylococcus aureus*. The cultures are incubated, and the inhibitory activity of the ICMP isolates monitored by the extent of reduction in bacterial light production compared to bacteria isolated with no fungus.

### 3.5. Antimicrobial Testing of Extracts

Dry samples of extracts were dissolved in DMSO to make a 25 mg/mL solution and then further diluted into Mueller Hinton broth II (MHB) to achieve a maximum concentration of 2 mg/mL. Each extract (200 µL) was added to two adjacent wells along the top of the 96-well plate (Thermo Fisher, NUN167008, Waltham, MA, USA). MHB (100 µL) was then added to the remaining wells and extract solution (100 µL) serially diluted two-fold down the plate and discarded. Aliquots of bacteria, *S. aureus* ATCC 29213 and *E. coli* ATCC 25922, at an optical density at 600 nm of 0.01 (approximately 1 × 10^6^ colony forming units (CFU)/mL) were then added to all the wells. This gave a maximum concentration of 1 mg/mL and a minimum concentration of 16 µg/mL. The maximum volume/volume concentration of DMSO in all extracts was 4%; therefore, the negative control was tested at an identical concentration.

Absorbance was measured at 600 nm using an Enspire plate reader (Perkin Elmer, MA, USA) at 0, 2, 4 and 20 h to determine the minimum inhibitory concentration (MIC), between which times the plates were incubated at 37 °C with shaking at 100 rpm. After 20 h, 10 µL of liquid from all wells showing inhibition of bacterial growth was pipetted onto a plate of MH agar. Once all liquid had evaporated, the plates were then incubated inverted at 37 °C for 16–20 h, and the minimum bactericidal concentration (MBC) was measured [5,17].

### 3.6. Antimicrobial Assays of Pure Compounds 

Bacterial strains *(S. aureus* ATCC 43300 (MRSA), *E. coli* ATCC 25922, *P. aeruginosa* ATCC 27853, *Klebsiella pneumoniae* ATCC 700603, *Acinetobacter baumannii* ATCC 19606) were cultured in either Luria broth (LB) (In Vitro Technologies, USB75852, Victoria, Australia), nutrient broth (NB) (Becton Dickson, 234,000, New South Wales, Australia) or MHB at 37 °C overnight [5,18]. A sample of culture was then diluted 40-fold in fresh MHB and incubated at 37 °C for 1.5−2 h. The compounds were serially diluted 2-fold across the wells of 96-well plates (Corning 3641, nonbinding surface), with compound concentrations ranging from 0.015 to 64 μg/mL, plated in duplicate. The resultant mid log phase cultures were diluted to the final concentration of 1 × 10^6^ CFU/mL; then, 50 μL was added to each well of the compound containing plates, giving a final compound concentration range of 0.008–32 μg/mL and a cell density of 5 × 10^5^ CFU/mL. All plates were then covered and incubated at 37 °C for 18 h. Resazurin was added at 0.001% final concentration to each well and incubated for 2 h before MICs were read by eye.

Fungi strains (*Candida albicans* ATCC 90028 and *Cryptococcus neoformans* ATCC 208821) were cultured for 3 days on YPD agar at 30 °C. A yeast suspension of 1 × 10^6^ to 5 × 10^6^ CFU/mL was prepared from five colonies. These stock suspensions were diluted with yeast nitrogen base (YNB) (Becton Dickinson, 233,520, New South Wales, Australia) broth to a final concentration of 2.5 × 10^3^ CFU/mL. The compounds were serially diluted 2-fold across the wells of 96-well plates (Corning 3641, nonbinding surface), with compound concentrations ranging from 0.015 to 64 μg/mL and final volumes of 50 μL, plated in duplicate. Then, 50 μL of a previously prepared fungi suspension, in YNB broth to the final concentration of 2.5 × 10^3^ CFU/mL, was added to each well of the compound-containing plates, giving a final compound concentration range of 0.008–32 μg/mL. Plates were covered and incubated at 35 °C for 36 h without shaking. *C. albicans* MICs were determined by measuring the absorbance at OD_530_. For *C. neoformans*, resazurin was added at 0.006% final concentration to each well and incubated for a further 3 h before MICs were determined by measuring the absorbance at OD_570–600_.

Colistin and vancomycin were used as positive bacterial inhibitor standards for Gram-negative and Gram-positive bacteria, respectively. Fluconazole was used as a positive fungal inhibitor standard for *C. albicans* and *C. neoformans*. The antibiotics were provided in 4 concentrations, with 2 above and 2 below their MIC value, and plated into the first 8 wells of Column 23 of the 384-well NBS plates. The quality control (QC) of the assays was determined by the antimicrobial controls and the Z’-factor (using positive and negative controls). Each plate was deemed to fulfil the quality criteria (pass QC), if the Z’-factor was above 0.4, and the antimicrobial standards showed full range of activity, with full growth inhibition at their highest concentration, and no growth inhibition at their lowest concentration [5,18]. 

### 3.7. Cytotoxicity Assays 

To a 384-well plate containing the 25× (2 μL) concentrated compounds, HEK-293 cells, counted manually in a Neubauer haemocytometer, were plated at a density of 5000 cells/well into each well [18,19]. The medium used was Dulbecco’s modified eagle medium (DMEM) supplemented with 10% fetal bovine serum (FBS). Cells were incubated together with the compounds for 20 h at 37 °C, 5% CO_2_. Resazurin (5 μL (equals 100 μM final)) was then added to each well and incubated for further 3 h at 37 °C with 5% CO_2_. After final incubation, fluorescence intensity was measured as Fex 560/10 nm, em 590/10 nm (F_560/590_) using a Tecan M1000 Pro monochromator plate reader. CC_50_ values (concentration at 50% cytotoxicity) were calculated by normalizing the fluorescence readout, with 74 μg/mL tamoxifen as negative control (0%) and normal cell growth as positive control (100%). The concentration-dependent percentage cytotoxicity was fitted to a dose–response function (using Pipeline Pilot) and CC_50_ values determined [18,19]. 

### 3.8. Haemolytic Assay 

Human whole blood was washed three times with 3 volumes of 0.9% NaCl and then resuspended in the same solution to a concentration of 0.5 × 10^8^ cells/mL, as determined by manual cell count in a Neubauer haemocytometer. The washed cells were then added to the 384-well compound-containing plates for a final volume of 50 μL. After a 10 min shake on a plate shaker, the plates were then incubated for 1 h at 37 °C. The plates were then centrifuged at 1000 g for 10 min to pellet cells and debris; 25 μL of the supernatant was then transferred to a polystyrene 384-well assay plate. Haemolysis was determined by measuring the supernatant absorbance at 405 mm (OD_405_) using a Tecan M1000 Pro monochromator plate reader. HC_10_ and HC_50_ (concentration at 10% and 50% haemolysis, respectively) were calculated by curve fitting the inhibition values vs. log(concentration) using a sigmoidal dose–response function with variable fitting values for top, bottom and slope [18,19].

## 4. Conclusions

A new unsymmetrical naphthoquinone dimer **1** was isolated from the organic extract of the mangrove endophytic fungus *Neofusicoccum australe* along with four other known natural products. Neofusnaphthoquinone B (**1**) exhibited moderate activity against methicillin-resistant *Staphylococcus aureus* (MRSA) with no detectable cytotoxicity or red blood cell haemolytic properties. These results identify **1** as a suitable candidate worthy of further investigation in antimicrobial drug discovery.

## Figures and Tables

**Table 1:** ^1^H and ^13^C-NMR data for compound **1** (DMSO-*d*_6_).

	δ_H_ (m, *J* in Hz) ^a^	δ_C_^b^	Selected HMBC Correlations
**1**		180.7	
**2**	-	159.6	
**3**	-	123.5	
**4**	-	189.9	
**4a**	-	108.6	
**5**	-	159.7	
**6**	-	126.6	
**7**	-	161.3	
**8**	7.11 (s)	102.1	1, 4a, 6, 7, 8a,
**8a**	-	129.6	
**9**	5.20 (dq, 7.0, 6.7)	60.6	5, 7, 10
**10**	1.41 (d, 6.7)	21.7	6, 9
**11**	3.92 (s)	56.2	7
**1′**	-	178.7	
**2′**	-	160.7	
**3′**	6.24 (s)	109.3	1′, 2′, 4′, 4a’
**4′**	-	190.3	
**4a′**	-	108.3	
**5′**	-	160.2	
**6′**	-	126.8	
**7′**	-	162.5	
**8′**	7.12 (s)	102.9	1′, 4a’, 6′, 7′, 8a’
**8a′**	-	130.0	
**9′**	4.80 (q, 7.0)	27.5	2, 4, 5′, 7′
**10′**	1.60 (d, 7.0)	17.6	3, 6′, 9′
**11′**	3.86 (s)	56.3	7′
**12′**	3.86 (s)	56.8	2′
**2-OH**	10.89 (br s)	-	
**5-OH**	13.38 (br s)	-	
**9-OH**	4.61 (d, 7.0)	-	9, 10
**5′-OH**	12.95 (s)	-	

^a^ Data recorded at 500 MHz. ^b^ Data recorded at 125 MHz.

**Table 2:** Antimicrobial and antifungal activities of **1**–**3** and **5**.

Compound	MIC (µg/mL)						
	*S. a* ^a^	*P. a* ^b^	*E. c* ^c^	*K. p* ^d^	*A. b* ^e^	*C. a* ^f^	*C. n* ^g^
**1**	16	>32 ^h^	>32 ^h^	>32 ^h^	>32 ^h^	>32 ^h^	>32 ^h^
**2**	16	>32 ^h^	>32 ^h^	>32 ^h^	>32 ^h^	>32 ^h^	>32 ^h^
**3**	>32 ^h^	>32 ^h^	>32 ^h^	>32 ^h^	>32 ^h^	>32 ^h^	>32 ^h^
**5**	>32 ^h^	>32 ^h^	>32 ^h^	>32 ^h^	>32 ^h^	>32 ^h^	>32 ^h^

All values are presented as the mean (*n* = 2). ^a^ *Staphylococcus aureus* ATCC 43300 (MRSA) with vancomycin (MIC 1 μg/mL) used as a positive control; ^b^ *Pseudomonas aeruginosa* ATCC 27853 with colistin (MIC 0.25 μg/mL); ^c^ *Escherichia coli* ATCC 25922 with colistin (MIC 0.125 μg/mL); ^d^ *Klebsiella pneumoniae* ATCC 700603 with colistin (MIC 0.25 μg/mL) as a positive control; ^e^ *Acinetobacter baumanii* ATCC 19606 with colistin (MIC 0.25 μg/mL) as a positive control; ^f^ *Candida albicans* ATCC 90028 with fluconazole (MIC 0.125 μg/mL) as a positive control; ^g^ *Cryptococcus neoformans* ATCC 208821 with fluconazole (MIC 8 μg/mL) as a positive control; ^h^ not active at a single dose test of 32 µg/mL.

**Table 3:** Cytotoxicity and haemolytic properties of **1** and **2**.

Compound	HEK-293 ^a^ CC_50_ (µg/mL)	HC_10_ (µg/mL) ^b^
**1**	>32 ^c^	>32 ^c^
**2**	>32 ^c^	>32 ^c^

All values presented as the mean (*n* = 2). ^a^ Concentration of compound at 50% cytotoxicity on HEK293 human embryonic kidney cells. Tamoxifen was the positive control (IC_50_ 9 μg/mL, 24 μM); ^b^ concentration of compound at 10% haemolytic activity on human red blood cells. Melittin was the positive control (HC_10_ 2.7 μg/mL); ^c^ not active at a single dose test of 32 µg/mL.

## Data Availability

Raw data for the antimicrobial activity testing is available at doi:10.17608/k6.auckland.11868675 and doi:10.17608/k6.auckland.11888184.

## Supplementary Materials

The following are available online. The ^1^H (Figure S1), ^13^C (Figure S2), COSY (Figure S3), HSQC (Figure S4), HMBC (Figure S5) and ROESY (Figure S6) NMR spectra, HRESIMS (Figure S7) and HPLC trace (Figure S8) for neofusnaphthoquinone B (**1**) and initial antibacterial screening results from zone of inhibition assays (Figure S9).

## Sample Availability

Samples of the compounds are available from the authors.
