# Supplementary material for: Antimicrobial Metabolites against Methicillin-Resistant Staphylococcus aureus from the Endophytic Fungus Neofusicoccum australe
Source: Molecules. 2021 Feb 19;26(4):1094. doi: 10.3390/molecules26041094 (PMC7922810; doi:10.3390/molecules26041094)
Supplement: Supplementary file 1 [file molecules-26-01094-s001.pdf]

Supporting Information

Antimicrobial metabolites against Methicillin-Resistant *Staphylococcus aureus* from the endophytic fungus *Neofusicoccum australe*

Melissa M. Cadelis*[1,2], Soeren G. Geese[2], Benedict B. Uy[2], Daniel R. Mulholland[2], Shara J. van de Pas[2], Alex Grey[2], Bevan S. Weir[3], Brent R. Copp[1†] and Siouxsie Wiles*[2†]

Addresses: [1]School of Chemical Sciences, University of Auckland, Private Bag 92019, Auckland 1142, New Zealand; [2]Bioluminescent Superbugs Lab, School of Medical Sciences, University of Auckland, Private Bag, 92019, Auckland 1142, New Zealand; [3]Manaaki Whenua/Landcare Research Ltd., Private Bag 92170, Auckland 1142, New Zealand.

Email: Melissa M. Cadelis – m.cadelis@auckland.ac.nz and Siouxsie Wiles – s.wiles@auckland.ac.nz

* Corresponding authors.

[†]These authors contributed equally to this work.

Content

Figure S1 ^{1}H NMR spectrum (500 MHz, DMSO) of neofusnaphthoquinone B (**1**) S2
Figure S2 ^{13}C NMR spectrum (125 MHz, DMSO) of neofusnaphthoquinone B (**1**) S3
Figure S3 COSY spectrum of neofusnaphthoquinone B (**1**) S4
Figure S4 HSQC spectrum of neofusnaphthoquinone B (**1**) S5
Figure S5 HMBC spectrum of neofusnaphthoquinone B (**1**) S6
Figure S6 ROESY spectrum of neofusnaphthoquinone B (**1**) S7
Figure S7 HRESIMS of neofusnaphthoquinone B (**1**) S8
Figure S8 HPLC trace of neofusnaphthoquinone B (**1**) S9
Figure S9 Antibacterial activity testing using the zone of inhibition (ZOI) assay S10

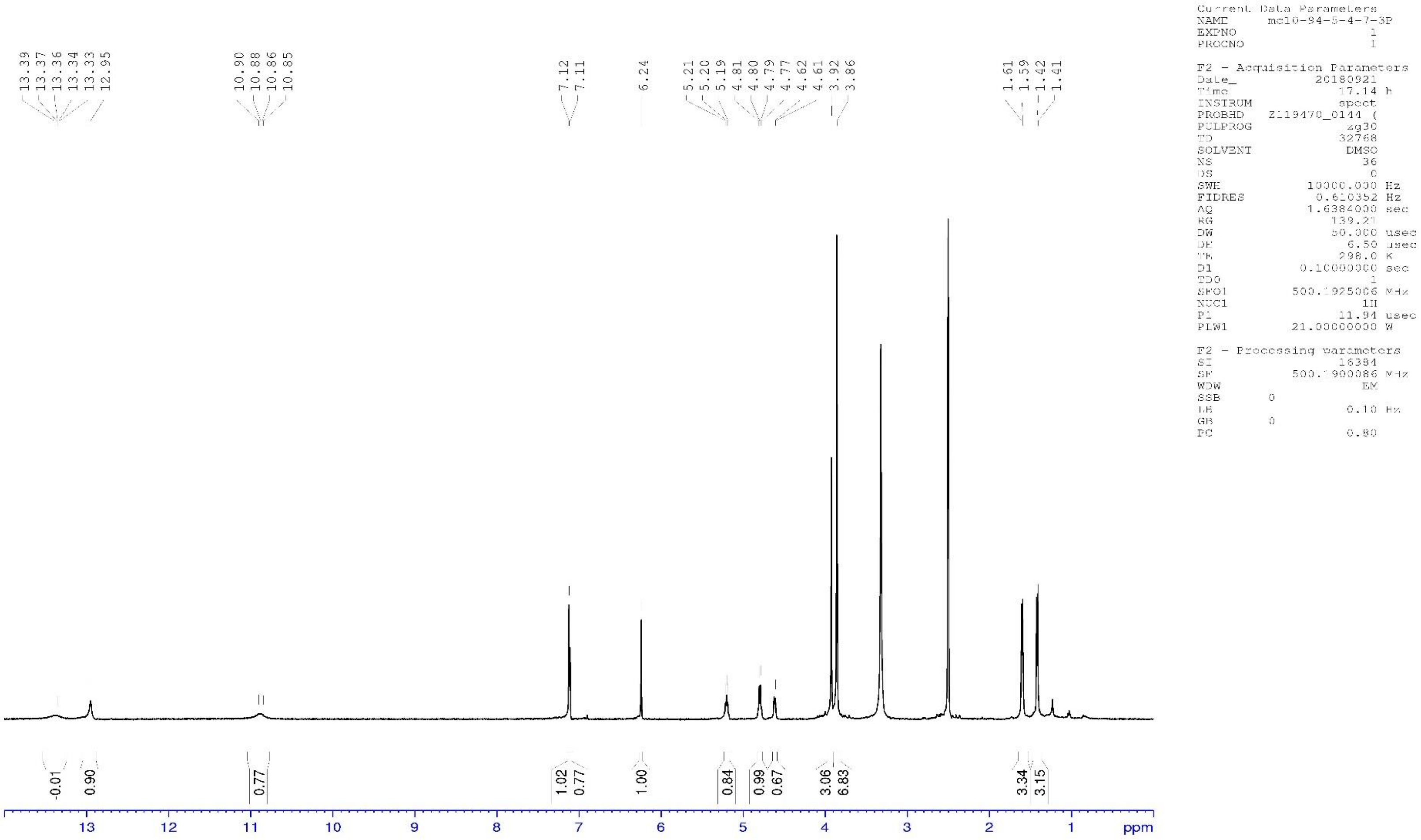

Figure S1 ¹H NMR spectrum (500 MHz, DMSO) of neofusnaphthoquinone B (**1**)

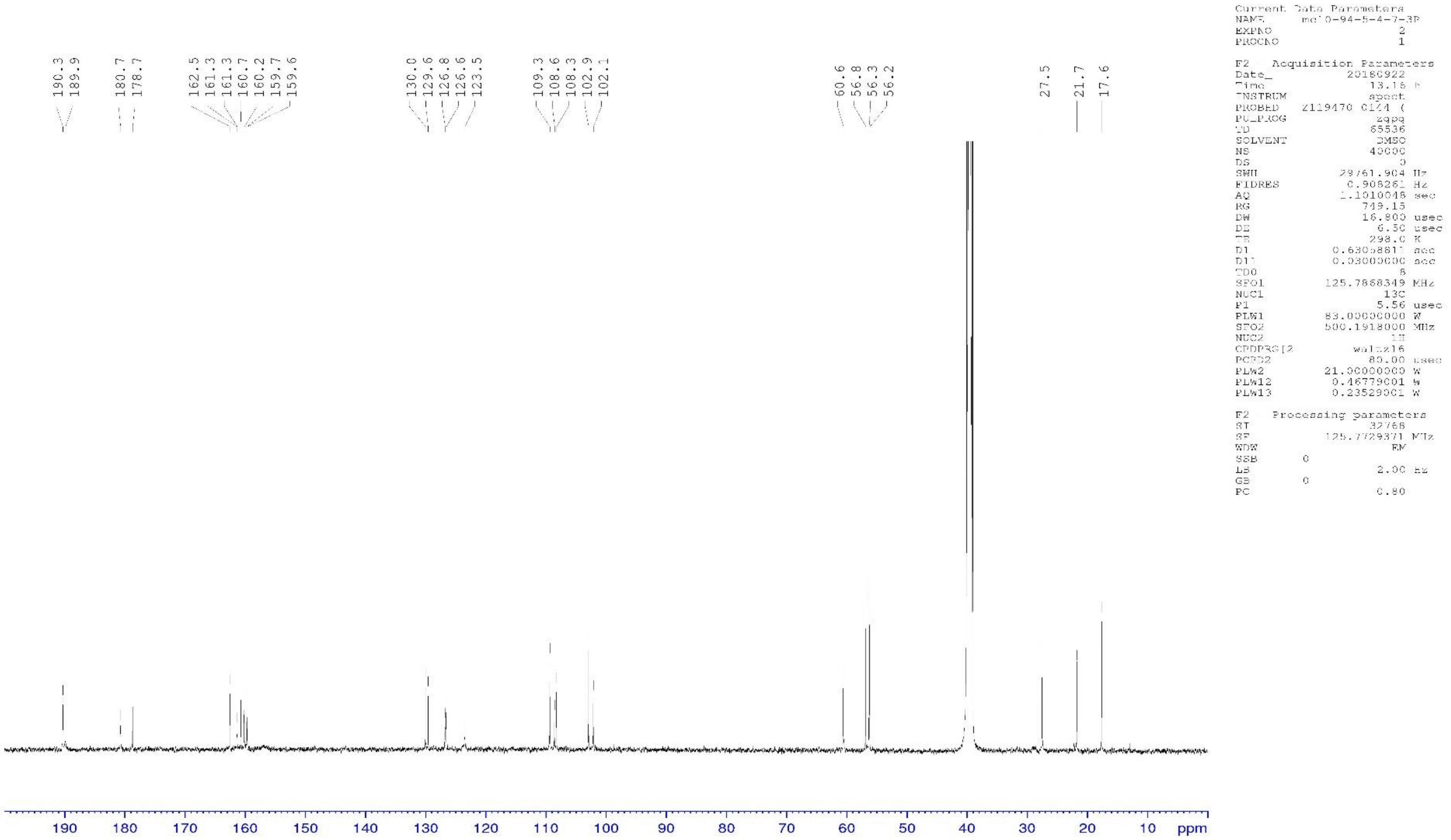

Figure S2 ^{13}C NMR spectrum (125 MHz, DMSO) of neofusnaphthoquinone B (**1**)

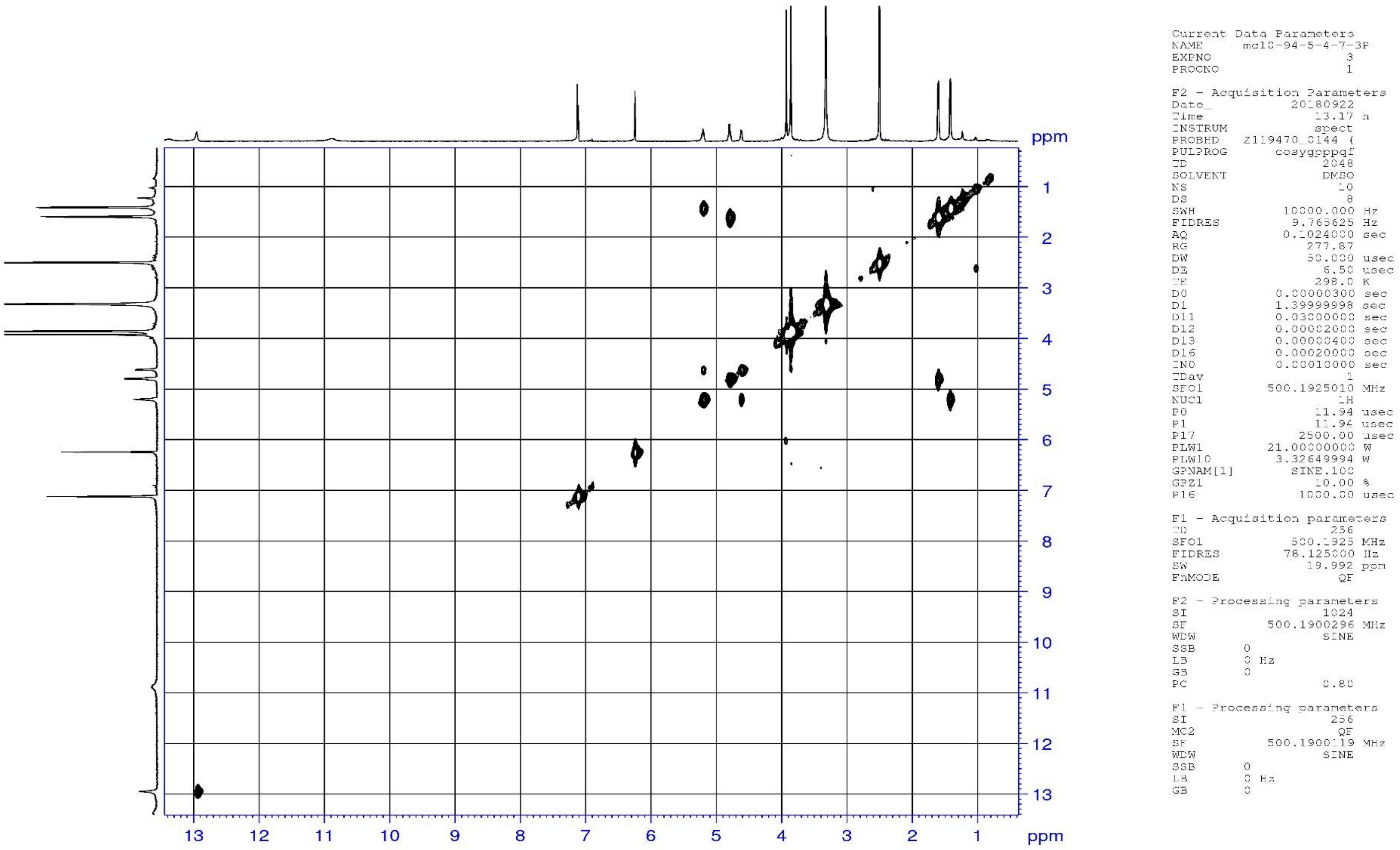

Figure S3 COSY spectrum of neofusnaphthoquinone B (**1**)

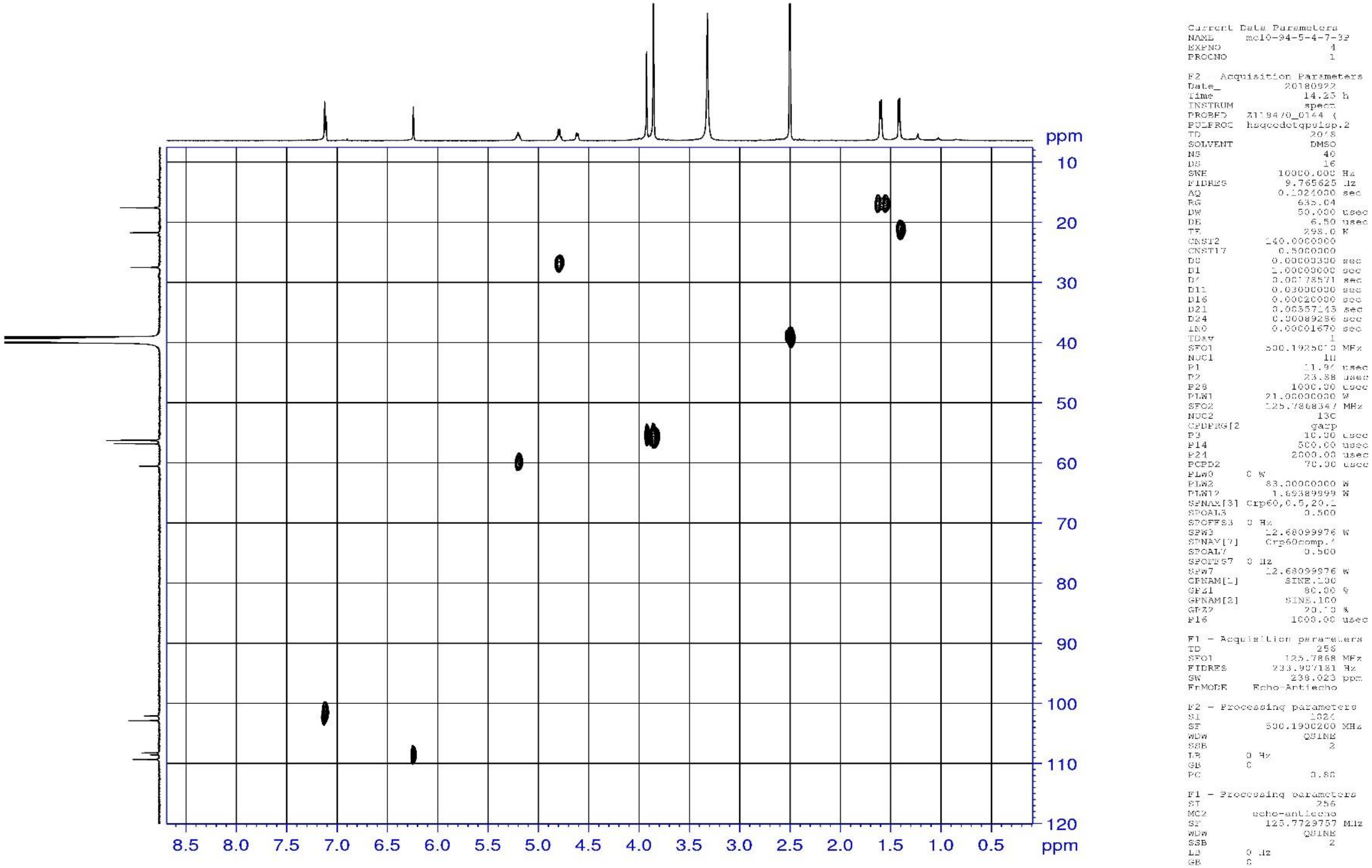

Figure S4 HSQC spectrum of neofusnaphthoquinone B (**1**)

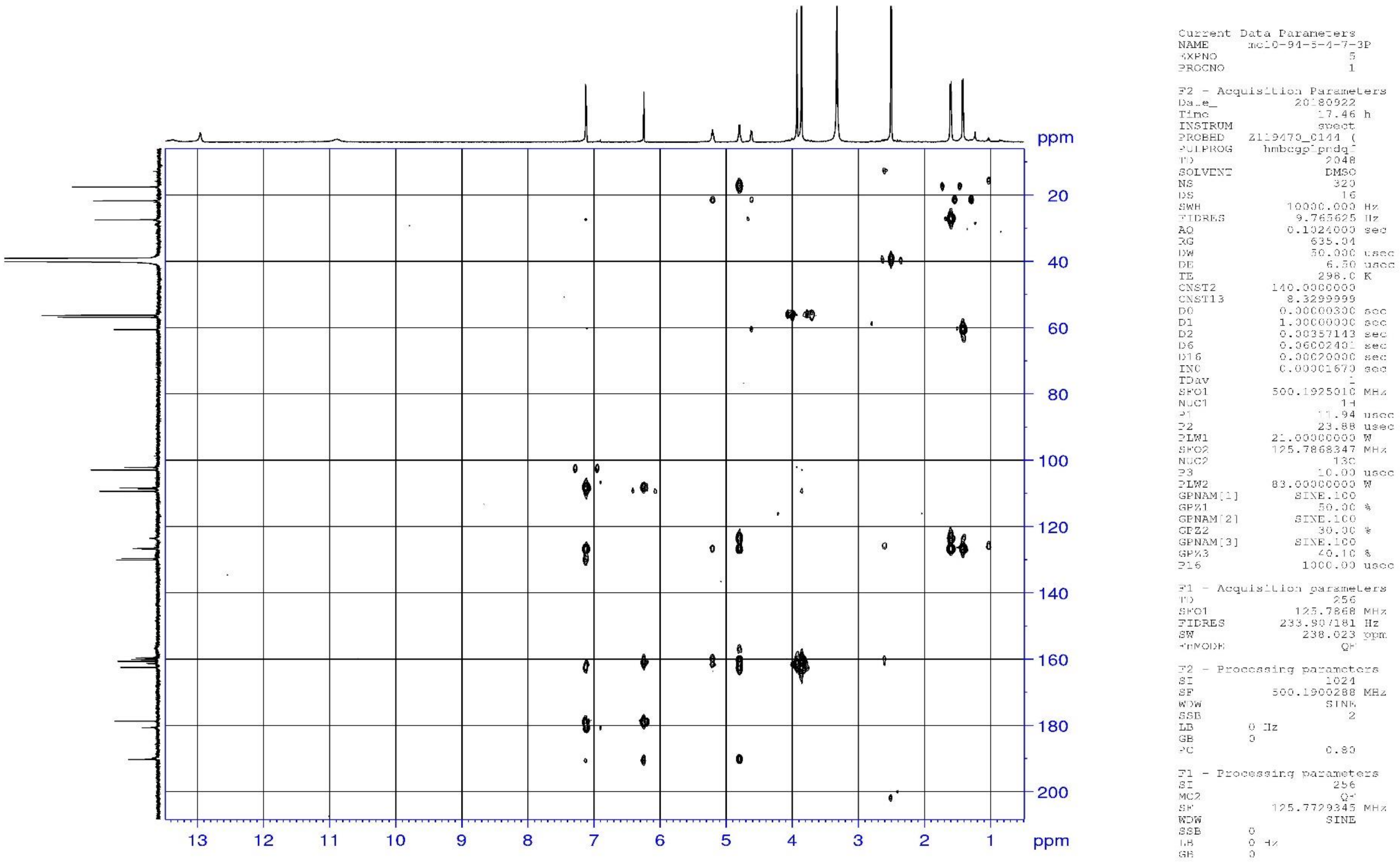

Figure S5 HMBC spectrum of neofusnaphthoquinone B (**1**)

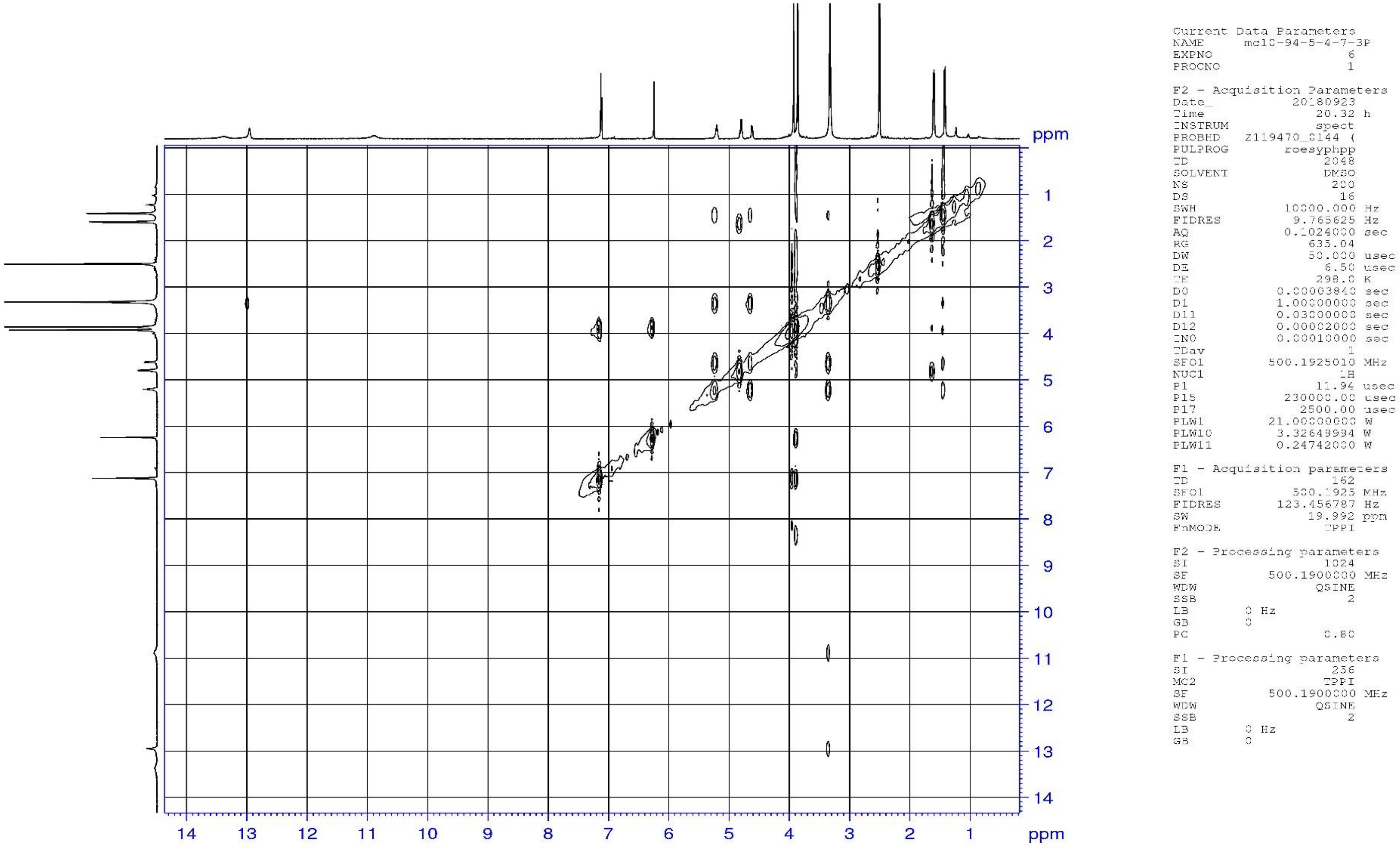

Figure S6 ROESY spectrum of neofusnaphthoquinone B (**1**)

Acquisition Parameter

Source Type	ESI	Ion Polarity	Positive	Set Nebulizer	0.4 Bar
Focus	Not active	Set Capillary	4500 V	Set Dry Heater	180 °C
Scan Begin	50 m/z	Set End Plate Offset	-500 V	Set Dry Gas	4.0 l/min
Scan End	1000 m/z	Set Collision Cell RF	150.0 Vpp	Set Divert Valve	Waste

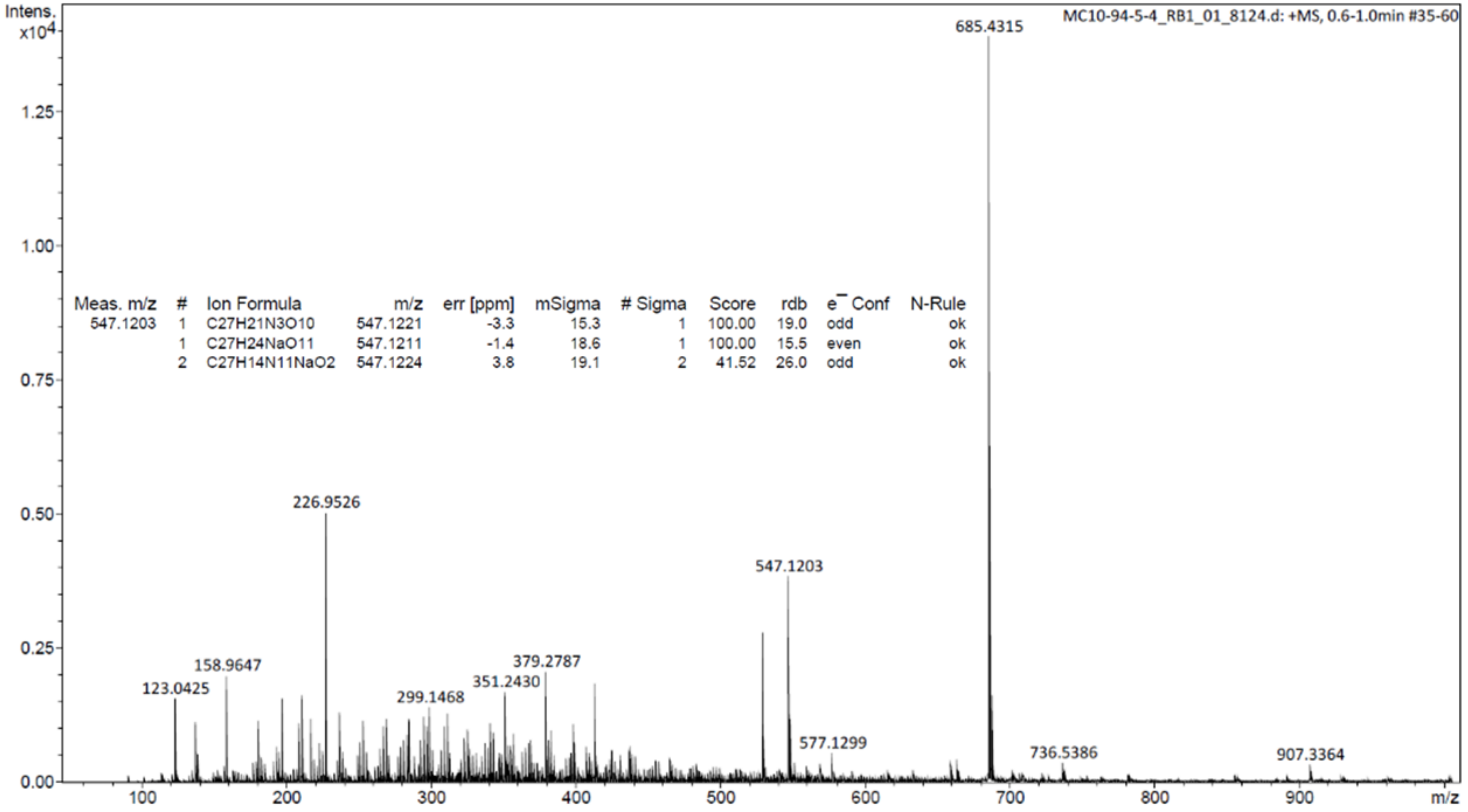

Figure S7 HRESIMS of neofusnaphthoquinone B (**1**)

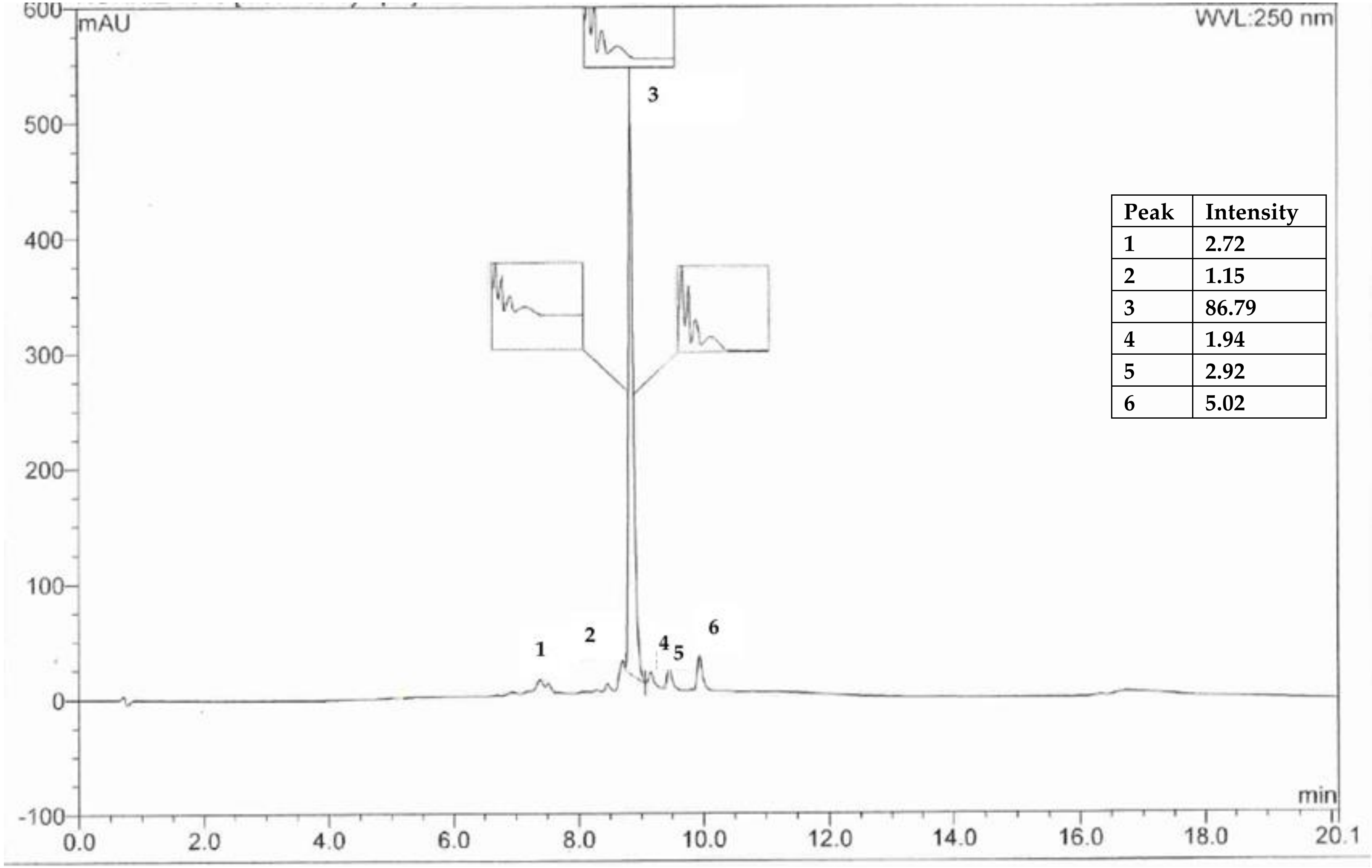

Peak	Intensity
1	2.72
2	1.15
3	86.79
4	1.94
5	2.92
6	5.02

Figure S8 HPLC trace of neofusnaphthoquinone B (**1**) (Analytical reversed-phase HPLC on a Dionex UltiMate 3000RS using a C_8 column (3 μm Platinum, 33 × 7 mm) and eluting with a linear gradient of H_2O (0.05% TFA) to MeCN over 20 mins at 2 mL/min and monitoring at 216 nm).

Figure S9 Antibacterial activity testing using the zone of inhibition (ZOI) assay

To perform zone of inhibition (ZOI) testing, potato dextrose agar (PDA) plates were inoculated with a lawn of either antibiotic-sensitive *Escherichia coli* (ATCC 25922) or resistant clinical isolates (CTX-M-9, CTX-M-14, CTX-M-15, NDM-1) or antibiotic-resistant *Klebsiella pneumoniae* (ATCC 700603). Similarly, Mueller-Hinton agar plates were inoculated with a lawn of either antibiotic-sensitive *Staphylococcus aureus* (ATCC 29213) or antibiotic-resistant *S. aureus* (ATCC 33593). *Neofusicoccum australe* was grown on PDA plates and fungal plugs removed using a 6 mm punch biopsy tool (Catalogue number: SH241, Amtech Medical, New Zealand). Fungal plugs were placed onto the bacterial lawns, alongside PDA plugs containing no fungus. Plates were incubated inverted at 37°C for 24 h before measuring any zones of inhibition (in mm) produced.

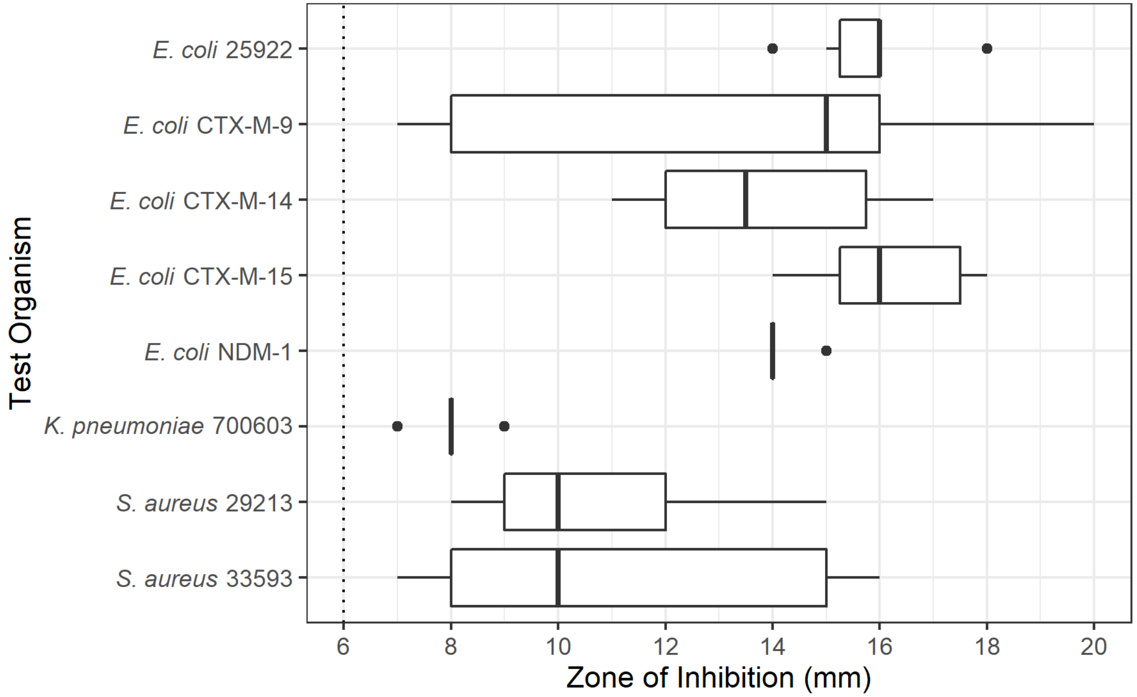

Antibacterial activity of *Neofusicoccum australe* (ICMP 21498) against *E. coli*, *K. pneumoniae*, and *S. aureus*.

The dotted line represents the diameter of the fungal plugs with no activity. Data is presented as box-whisker plots. Raw data is available at DOI: 10.17608/k6.auckland.11888184.
